# Dynamic Changes in Plasma Metabolic Profiles Reveal a Potential Metabolite Panel for Interpretation of Fatal Intoxication by Chlorpromazine or Olanzapine in Mice

**DOI:** 10.3390/metabo12121184

**Published:** 2022-11-27

**Authors:** Rui Bai, Xiaohui Dai, Xingang Miao, Bing Xie, Feng Yu, Bin Cong, Di Wen, Chunling Ma

**Affiliations:** 1 Hebei Key Laboratory of Forensic Medicine, Collaborative Innovation Center of Forensic Medical Molecular Identification, College of Forensic Medicine, Hebei Medical University, Shijiazhuang 050017, China; 2 Research Unit of Digestive Tract Microecosystem Pharmacology and Toxicology, Chinese Academy of Medical Sciences, Shijiazhuang 050017, China; 3 Forensic Science Centre of WATSON, Guangzhou 510440, China

**Keywords:** fatal intoxication, chlorpromazine, olanzapine, metabolomics analysis, LC-MS, chemometrics

## Abstract

Diagnosing the cause of fatal intoxication by antipsychotic agents is an important task in forensic practice. In the 2020 Annual Report of the American Association of Poison Control Centers, among 40 deaths caused by antipsychotics, 21 cases were diagnosed as “probably responsible”, thereby indicating that more objective diagnostic tools are needed. We used liquid chromatography-mass spectrometry-based integrated metabolomics analysis to measure changes in metabolic profiles in the plasma of mice that died from fatal intoxication due to chlorpromazine (CPZ) or olanzapine (OLA). These results were used to construct a stable discriminative classification model (DCM) comprising L-acetylcarnitine, succinic acid, and propionylcarnitine between fatal intoxication caused by CPZ/OLA and cervical dislocation (control). Performance evaluation of the classification model in mice that suffered fatal intoxication showed relative specificity for different pharmacodynamic drugs and relative sensitivity in different life states (normal, intoxication, fatal intoxication). A stable level of L-acetylcarnitine and variable levels of succinic acid and propionylcarnitine between fatal-intoxication and intoxication groups revealed procedural perturbations in metabolic pathways related to fatal intoxication by CPZ/OLA. Additional stability studies revealed that decomposition of succinic acid in fatal-intoxication samples (especially in the OLA group) could weaken the prediction performance of the binary-classification model; however, levels of these three potential metabolites measured within 6 days in fresh samples kept at 4 °C revealed a good performance of our model. Our findings suggest that metabolomics analysis can be used to explore metabolic alterations during fatal intoxication due to use of antipsychotic agents and provide evidence for the cause of death.

## 1. Introduction

The 2020 Annual Report of the American Association of Poison Control Centers showed that the most overdose fatalities were caused by sedative/hypnotic/antipsychotic medication (fatal intoxication), and the numbers have continued to increase in the past decade [1]. Usually, antipsychotic agents are prescribed for the treatment of severe mental disorders such as schizophrenia and bipolar disorder because of their sedative effects; these drugs are also employed to treat anxiety, depression, and drug dependence [2], and they are used commonly in drug-related suicides [3]. According to epidemiological data, typical antipsychotics such as chlorpromazine (CPZ), perphenazine, and atypical antipsychotics such as olanzapine (OLA) and clozapine are among the antipsychotics associated with a high prevalence of fatality in the past decade [4,5,6].

In general, identification of the cause of death from acute overdose of an antipsychotic agent involves determining the blood concentration of the drug and intoxication symptoms. For different antipsychotics, cardiometabolic issues are associated with the lifespan to varying degrees [7], which make the cause of death complicated. In addition, owing to the instability of common antipsychotics (e.g., OLA) in stored blood specimens [8] and large inter-individual variability with respect to first-pass metabolism by the liver (e.g., CPZ) [9], the reference intoxication or lethal drug levels in blood can be used only as orientating factors [10,11]. Contents of antipsychotics in blood can indicate if an overdose occurred [12]. However, compared with exogenous metabolites whose blood content were mainly influenced by liver/kidney functions, endogenous molecules involved in pathological processes (especially in the acute-phase response after overdose) can be relatively specific and used for quality-of-life monitoring [13].

The endogenous metabolome is highly conserved as measured across species [14]. Metabolomics analysis (MA) is suitable for the identification and screening of the metabolites used for the diagnosis of fatal intoxication by antipsychotic agents [15,16]. Based on this method, some perturbed metabolic pathways, such as fatty-acid oxidation [17,18] and the tricarboxylic acid (TCA) cycle [19], have been reported to be related to antipsychotic treatment. However, what happens to blood metabolism when death occurs is not known. For differential metabolites related to the above perturbed metabolic pathways, Meister et al. showed that complementary treatment with L-acetylcarnitine led to a lower risk of experiencing any adverse event associated with antipsychotics or other psychotropic drugs, such as tricyclic antidepressants and selective serotonin reuptake inhibitors [20]. Hence, more than one potential endogenous metabolite is needed for the interpretation of lethal poisoning by antipsychotic agents. As reported by Jordan and colleagues, diagnostic chemicals should be significantly causally/mechanistically related with forensic endpoints, highly sensitive and specific, and reproducible [21]. For forensic diagnoses, the changes in chemicals pre mortem and chemical stability post mortem are also important.

In the present study, a liquid chromatography-mass spectrometry (LC-MS)-based untargeted metabolomics tool was used for metabolic-pathway analysis in plasma samples after fatal intoxication due to CPZ or OLA. Moreover, a potential metabolite panel was fitted in a discriminative classification model (DCM). The latter divides samples into different categories and predicts the labels of samples through a series of important features [22]. Targeted measurements of potential metabolites were utilized further under a multiple reaction monitoring (MRM) model. Associated specificity, sensitivity, and stability evaluations were conducted at different drug levels and life states.

## 2. Materials and Methods

### 2.1. Chemicals

Methanol, acetonitrile, and formic acid (grade: high-performance liquid chromatography) were purchased from Dikma Technology (Foothill Ranch, CA, USA). Deionized water was purified by the Milli-Q™ system (Millipore, Burlington, MA, USA). CPZ, OLA, clozapine, perphenazine, promethazine, and estazolam were obtained from Dalian Meilune Biotechnology (Dalian, China). The standard for L-acetylcarnitine (US Chemical Abstracts Service (CAS) number: 3040-38-8) was sourced from Shanghai APExBIO Biological Technology (Shanghai, China). Standards for succinic acid (CAS number: 110-15-6), propionylcarnitine (20064-19-1), and their associated stable isotope-labeled internal standards (ISs) L-acetylcarnitine-(N-methyl-d3) (204259-54-1), propionylcarnitine-(N-methyl-d3) (203806-01-3), and succinic acid-2,2,3,3-d4 (14493-42-6) for targeted MA were purchased from Mil-liporeSigma (Burlington, MA, USA).

### 2.2. Experimental Animals

CD1 mice (7–8 weeks; female: 32–37 g; male: 38–42 g) were housed in a room at 23 ± 2 °C with relative humidity of 55 ± 5% and a standard 12-h dark–light cycle. Mice were fed with specific pathogen free-grade chow for 1 week and fasted overnight (with ad libitum access to water) before experimentation.

All drug concentrations were adjusted to an appropriate volume for intragastric administration (0.2–0.35 mL) immediately before use. Doses differed according to experimental design.

The high doses for fatal intoxication using CPZ and OLA were 150 mg/100 g (5-fold median lethal dose (LD_50_) = 30 mg/100 g) and 80 mg/100 g (4-fold LD_50_ (20 mg/100 g)), respectively. The high dose for the fatal-intoxication groups of clozapine, perphenazine, promethazine, and estazolam were 80 mg/100 g (4-fold LD_50_), 150 mg/100 g (6-fold LD_50_), 125 mg/100 g (5-fold LD_50_), and 600 mg/100 g (10-fold LD_50_), respectively. These doses were chosen according to the literature [23,24,25,26] and our protocol. The low dose for the fatal-intoxication and intoxication groups of CPZ and OLA was 2-fold LD_50_ and LD_50_; and the therapeutic dose was 0.375 mg/100 g and 0.15 mg/100 g [27,28,29], respectively. Mice in control groups were scarified by cervical dislocation. Mixed mice (males and females) were number-balanced in each group. A total of 127 animals were tested for untargeted MA (five intoxications were not included at a high dose treated by CPZ/OLA). For targeted MA, an additional 60 mice were tested (three intoxications were not included at a high dose treated by CPZ/OLA). Group information is summarized in Table 1.

### 2.3. Collection of Blood Samples

For all animals, blood from the abdominal aorta was collected immediately after cervical dislocation (controls, therapeutic animals, and intoxications) or death (fatal intoxications). For therapeutic groups, animals were killed 2 h after final administration of the drug. A paired sampling method was used for intoxications and fatal intoxications at a low dose. First, samples were transferred to heparin-treated 1.5-mL centrifuge tubes and centrifuged twice at 8000× *g* for 10-min each at 4 °C. The supernatant (blood plasma) was collected, snap-frozen in liquid nitrogen, and stored at −80 °C until use. For stability evaluation, we used plasma samples stored at 4 °C for 10 days; 80 μL of plasma was absorbed on days 0, 2, 4, 6, 8, and 10, and re-stored at −80 °C before processing.

### 2.4. Extraction of Metabolites from Blood

Plasma for untargeted MA was processed according to the methods described by Xia and colleagues [30]. Briefly, a plasma sample (100 μL) was added to a tube containing ice-cold methanol (300 μL). Each sample was vortex-mixed for 30 s, sonicated for 10 min in an ice-water bath, and incubated for 20 min at −20 °C to precipitate proteins. Then, the mixture was centrifuged at 12,000× *g* for 10 min at 4 °C. The resulting supernatant was transferred to a LC-MS vial for analysis. A quality control (QC) sample was prepared by mixing an aliquot of equal volume of the supernatant from each sample.

A version of the method described by Xia and colleagues [30] and modified by Roy and coworkers [31] was used for targeted MA. Briefly, 10 μL of an IS working solution was mixed with an aliquot of plasma (100 μL). Then, an aliquot of methanol (290 μL) was added, the sample was vortex-mixed, and the solution was kept for 20 min at −20 °C. Next, the sample was centrifuged at 12,000× *g* for 10 min at 4 °C. The supernatant was collected and evaporated to dryness under nitrogen and reconstituted with Milli-Q water (200 μL) for analysis.

Stock solutions of each standard (1 mg/mL) were prepared by dissolving neat compounds in methanol. Working solutions were created by addition of the appropriate volume of stock solution to Milli-Q water to yield a final analyte concentration of 10 μg/mL for L-acetylcarnitine, 1 μg/mL for propionylcarnitine, and 10 μg/mL for succinic acid. A plasma sample free of these three standards was not available, so we prepared calibration curves in Milli-Q water. A mixture comprising 490 μL of L-acetylcarnitine working solution, 262 μL of propionylcarnitine working solution, 561.6 μL of succinic acid working solution, and 686.4 μL of Milli-Q water was diluted serially to generate the calibration curve. For each time point, a 190-μL aliquot of the appropriate dilution was transferred to a chromatographic tube and mixed with an aliquot (10 μL) of labeled IS. Neat IS was dissolved in methanol to yield stock solutions (1 mg/mL) that were kept at 4 °C. Working solutions were obtained by adding the appropriate volume of stock solutions to Milli-Q water to yield a final analyte concentration of 1.5 μg/mL for L-acetylcarnitine-(N-methyl-d3), 1.5 μg/mL for propionylcarnitine-(N-methyl-d3), and 20 μg/mL for succinic acid-2,2,3,3-d4.

### 2.5. Data Acquisition with Full Scan-MS/MS Using Liquid Chromatography-High Resolution Tandem Mass Spectrometry (LC-HR-MS/MS)

Spectra were calibrated according to mass internally by continuous infusion of a reference mass solution. The core methodologies of untargeted and targeted MA were based on a protocol [32]. Briefly, using the UPLC HSS T3 column (2.1 × 100 mm, 1.8 μm), LC-MS/MS was undertaken using the UltiMate™ 3000 (Thermo Fisher Scientific, Waltham, MA, USA) UPLC setup coupled with a mass spectrometer (Q Exactive; Thermo Fisher Scientific, Waltham, MA, USA). Mobile phase A was 0.1% formic acid in Milli-Q water. Mobile phase B was acetonitrile. The elution gradient was set as follows: 0 min, 98% A; 1 min, 98% A; 12 min, 2% A; 16 min, 2% A; 16.1 min, 98% A; 20 min, 98% A. The flow rate was 0.3 mL/min. The injection volume was 5 μL. During LC/MS, the mass spectrometer was used to acquire MS/MS spectra on an information-dependent basis. Depending on preselected criteria, the acquisition software (Xcalibur 4.0.27; Thermo Fisher Scientific) evaluated the full-scan survey MS data continuously when it collected and triggered the acquisition of MS/MS spectra in this mode. The conditions for the electrospray ion (ESI) source were set as follows: flow rate of sheath gas = 35 psi; flow rate of auxiliary gas = 15 Arb; capillary temperature = 350 °C; full MS resolution = 35,000; MS/MS resolution = 17,500; and data points = five. The collision energy was 25/35/45 eV in the normalized collision-energy model, and the spray voltage was 3.2 kV for positive polarity mode and −3.1 kV for negative polarity mode.

### 2.6. MRM Analysis by LC-MS/MS

A rapid resolution liquid chromatograph (ExionLC™ AC; SCIEX, Framingham, MA, USA) was coupled to a 5500 Q-Trap system (SCIEX) with an ESI source. Chromatographic separation was undertaken on a HSS C18 column (2.1 × 100 mm, 1.8 μm; Waters, Milford, MA, USA) at 35 °C. The flow rate was 0.25 mL/min. The injection volume was 2 μL. Mobile phase A was 0.01% formic acid with ammonium acetate (0.1 mM) in Milli-Q water. Mobile phase B was acetonitrile. The linear gradient was the same as that described for untargeted MA (equilibrium duration = 16.1–20 min, 98% A). The Q-Trap system was operated in positive and negative ion modes using MRM. The source-dependent parameters were ion spray voltage = 5 kV (−4.5 kV for negative); vaporizer temperature = 500 °C, nebulizing gas (GS1) = 40 psi; drying gas (GS2) = 45 psi; curtain gas = 30 psi. Acquisition and processing of data were done using Analyst 1.6 (SCIEX).

### 2.7. Data Processing and Statistical Analysis for Untargeted MA

Files for raw MS data were processed by Compound Discoverer 3.1 (Thermo Fisher Scientific). Low-quality data were filtered using the minimum signal/noise threshold (3) for each centroid. Other peak-picking parameters such as retention time (RT) width (0.2 min), mass tolerance (5 ppm), and minimum peak intensity (600,000) were set to default. QC samples were utilized for corrections of the compound area. Based on this strategy, features with a relative standard deviation >30% or coverage <50% for the areas for a particular compound across QC samples were also abandoned. To further improve the accuracy for feature identification in targeted MA, only features with at least one data-dependent MS2 scan for the preferred adduct ion identified by the mzCloud (https://www.mzcloud.org/ (accessed on 30 September 2021)) were calculated [33].

All multivariate analyses were conducted with MetaboAnalyst 5.0 (www.metaboanalyst.ca/ (accessed on 28 January 2022)). Normalization of data was carried out by autoscaling (mean-centering and division by the standard deviation of each variable) and log10-transformation. Principal component analysis (PCA) and quantitative enrichment analysis (QEA) were conducted to inspect differences in metabolite profiles visually between the fatal-intoxication CPZ/OLA groups and controls. Pathway Impact (PI) score >0 (using analysis of network topology) and *p* < 0.05 (using QEA) were considered to be potential primary pathways. The results for Student’s *t*-tests were combined with orthogonal projections to latent structures discriminant analysis (OPLS-DA) for screening of differential metabolites. Only those that met the significance criterion and participated in metabolic pathways were used further for construction of panels of differential metabolites between fatal-intoxication and control groups. Monte Carlo cross-validation based on the linear Support Vector Machine algorithm was used for evaluation of performance for discriminative differential metabolites. The area under the receiver operating characteristic (ROC) curve (AUC) and K-means clustering results were used to determine the choice of metabolite panels for DCM construction. Usually, a combination of different-behavior metabolites with the AUROC closer to 1 can show a better discriminative performance [34]. Permutation tests were carried out to ascertain the stability and robustness of our classification model [35]. A PCA bi-plot adds variable weights to show their influence upon sample clustering.

### 2.8. Statistical Analyses for Targeted MA

Quantitative and statistical analyses of L-acetylcarnitine, succinic acid, and propionylcarnitine were conducted with Prism 6 (GraphPad, San Diego, CA, USA). To compare the levels of three potential metabolites between different groups (fatal intoxication and intoxication, high and low doses), we carried out Student’s *t*-tests or Mann–Whitney rank sum tests for unpaired samples. Values are expressed as the mean ± standard deviation or median (25–75% percentile) according to normality as verified by D’Agostino and Pearson omnibus normality tests. Differences were considered significant at *p* < 0.05. For evaluation of the stability of the three potential metabolites within 10 days, “analyte instability” was defined as a deviation ≥15% from that on day 0. Values are expressed as the mean.

## 3. Results

### 3.1. Symptoms of Intoxication

For intoxication and fatal-intoxication groups treated by CPZ/OLA at low or high doses using a one-time administration method, animals first developed a resting tremor, followed by dyskinesia, and ~80% of animals that suffered fatal intoxication showed intense involuntary movement ~10 min before death. The time-course for all animals that suffered fatal intoxication was <12 h. Animals treated by CPZ showed a longer time-course (20–120 min) compared with those treated by OLA (15–60 min) at a high dose, and a longer time-course (3–6 h) compared with those treated with OLA (1–3 h) at a low dose. Animals treated by a therapeutic dose showed only mild sedative symptoms compared with controls.

### 3.2. Overall MA

**Figure 1:**
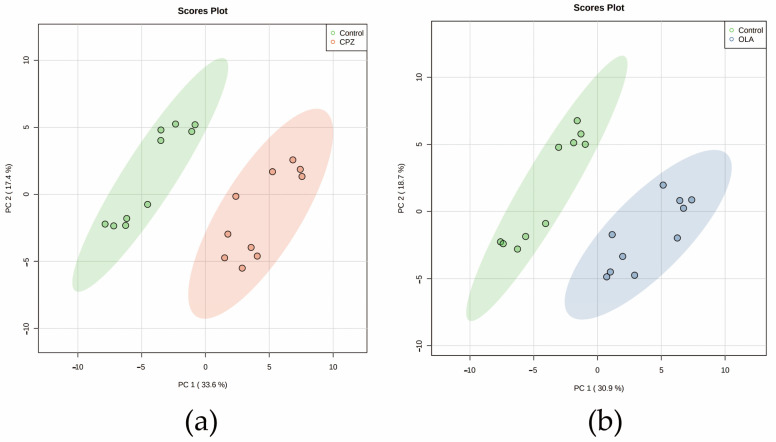
Plots of PCA score for metabolic profiling of the fatal-intoxication animal model. (**a**) CPZ and (**b**) OLA fatal intoxication vs. controls. *n* = 10. Abbreviations: CPZ: fatal intoxication by CPZ at a high dose; OLA: fatal intoxication by OLA at a high dose.

We focused on changes in metabolic profiles between CPZ/OLA fatal-intoxication (high dose, *n* = 10, respectively) and control (*n* = 10) groups. A total of ~8000 features were discovered through peak processing, and ~3000 compounds remained after grouping by molecular weight and RT. Among these compounds, 74 features with verified MS2 values in the mzCloud database were subjected to PCA to obtain a direct overview of systemic alterations in metabolites. PCA score plots showed distinct metabolic patterns between fatal-intoxication and control groups (Figure 1a,b). Combination of the results of Student’s *t*-tests with OPLS-DA revealed 28 and 29 differential metabolites to be screened out and classified according to the Metabolomics Standard Initiative [36] for fatal intoxication by CPZ and OLA, respectively (Table 2 and Table 3).

### 3.3. Analysis of Perturbed Metabolic Pathways

**Figure 2:**
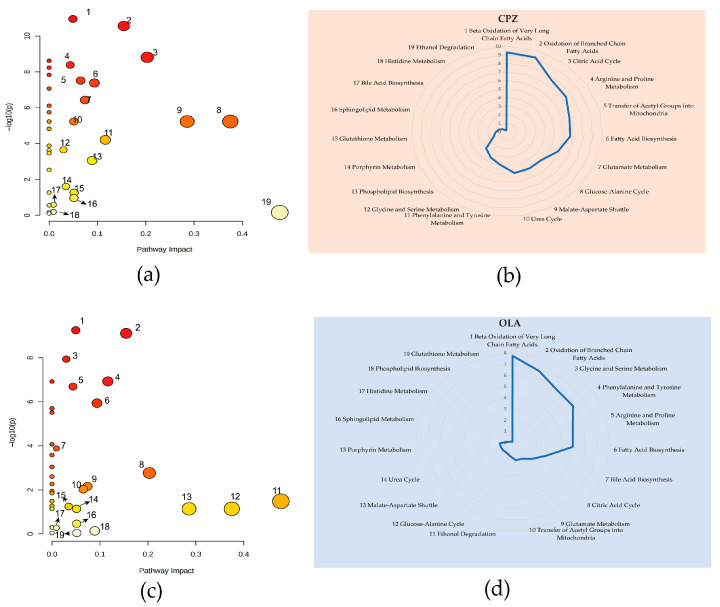
QEA results of fatal-intoxication models due to CPZ (**a**,**b**) and OLA (**c**,**d**) at high dose. Nineteen metabolic pathways with a PI score >0 in the CPZ animal model (**a**) and their associated logarithmic-transformed *p*-values (**b**). Nineteen metabolic pathways with a PI score >0 in the OLA animal model (**c**) and their associated logarithmic-transformed *p*-values (**d**). *n* = 10. Notes: Pathways represented by bubbles in a and c with a PI score >0 are listed by numbers according to their *p* (log-*p*) values. Radar charts were utilized to show the names of metabolic pathways, and their significance is marked with the same numbers as those in bubble plots. Arrows in bubble plots are used to reduce the crowding of numeric markers.

We wished to avoid screening of compounds which had occasional deviations in levels. Hence, we calculated only the differential metabolites that participated in metabolic pathways and were recorded in Small Molecule Pathway Database (SMPD; www.smpdb.ca/ (accessed on 5 January 2022)) and Kyoto Encyclopedia of Genes and Genomes (KEGG; www.genome.jp/ (accessed on 5 January 2022)) databases. QEA results revealed 19 identical metabolic pathways using a PI score >0 in CPZ and OLA fatal-intoxication groups (Figure 2a,b). Among these pathways, “Beta Oxidation of Very Long Chain Fatty Acids”, “Oxidation of Branched Chain Fatty Acids”, “TCA cycle”, “Fatty Acid Biosynthesis”, and “Arginine and Proline Metabolism” were the most significant according to a Holmes-adjusted *p*-value (log-*p*) (Figure 2c,d). Among these 28/29 differential metabolites of CPZ/OLA groups, seven common differential metabolites (L-acetylcarnitine, succinic acid, L-carnitine, propionylcarnitine, 3-oxotetradecanoic acid, citric acid, and R-3-hydroxydecanoic acid) were associated with these common perturbed metabolic pathways.

### 3.4. Construction of a DCM

**Figure 3:**
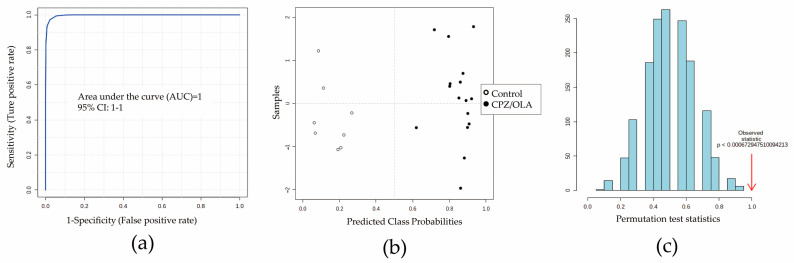
Evaluation of potential combinations of metabolites by a training set. (**a**) The area under the ROC curve (AUC) for prediction performance was 1 (95%CI of AUC: 1–1). (**b**) Predicted class probabilities between CPZ/OLA fatal intoxications (high dose) and controls. (**c**) Permutation tests showing the DCM to be outside the distribution of random class assignments based on each respective between-group sum of the squares and within-group sum of squares (B/W) ratio (*p* < 0.05). *n* = 8.

Before identifying differential metabolites, seven important metabolites were identified further by ion-trap detection based on their MS/MS information, and four differential metabolites (L-acetylcarnitine, succinic acid, L-carnitine, and propionylcarnitine) could be verified by the MRM model according to MS/MS data obtained from full-scan detection (Figure S1). Detection information (RT, parent ion, fragment ion, declustering potential, and collision energy) is shown in Table S1. Next, multivariate analysis based on ROC curves was undertaken to develop a better DCM. The predictive performances of each metabolite are shown in Table S1. A combination of L-acetylcarnitine, succinic acid, and propionylcarnitine showed a good prediction performance (*n* = 8; AUC = 1 [95% confidence interval (CI) of AUC: 1–1]) (Figure 3a,b). The average accuracy based on 100 cross-validations was 1. The results of permutation tests (*p* < 0.05) confirmed the stability and robustness of the DCM (Figure 3c). A validation set containing six (n_fatal intoxication_ = four, n_control_ = two) new samples for external validation of the DCM also showed 100% prediction accuracy (Table S2).

### 3.5. Specificity Evaluation of the Panel of Potential Metabolites

**Figure 4:**
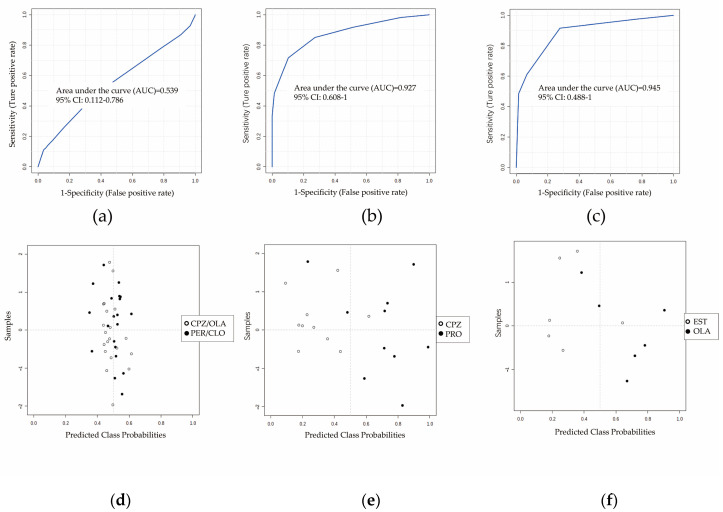
Differential performance of combinations of L-acetylcarnitine, succinic acid, and propionylcarnitine between antipsychotic groups and other groups of therapeutic drugs that led to fatal intoxication. ROC curves (**a**–**c**) and predicted class probabilities (**d**–**f**) show the differential performance and misclassification probabilities between antipsychotics and other therapeutic drugs. Discrimination with clozapine and perphenazine (**a**,**d**) was poor (AUC: 0.5; 95%CI of AUC: 0.1–0.8; *n* = 10), whereas that with promethazine (**b**,**e**; AUC: 0.9; 95%CI of AUC: 0.6–1; *n* = 10) or estazolam (**c**,**f**; AUC: 0.9; 95%CI of AUC: 0.5–1; *n* = 6) was better. Abbreviations: CLO: clozapine at a high dose that caused fatal intoxication; PER: perphenazine at a high dose that caused fatal intoxication; PRO: promethazine at a high dose that caused fatal intoxication; EST: estazolam at a high dose that caused fatal intoxication.

We wished to further verify the specificity of the DCM. Four sets of fatal-intoxication animal models treated using two drugs with similar pharmacodynamic properties (perphenazine and clozapine (antipsychotics), *n* = 10) and chemical properties (promethazine (phenothiazine, *n* = 10) and estazolam (benzodiazepines, *n* = 6)) as CPZ or OLA (*n* = 10) were introduced as negative groups. The performance among the four antipsychotics was poor (AUC = 0.5; 95%CI of AUC: 0.1–0.7) (Figure 4a,d) but that of promethazine (AUC = 0.9; 95%CI of AUC: 0.6–1) (Figure 4b,e) and estazolam was better (AUC = 0.9; 95%CI of AUC: 0.5–1) (Figure 4c,f).

### 3.6. Dynamic Changes of Three Potential Metabolites at Different Drug Levels

As the drug level increased, a similar tendency in levels of three potential metabolites between CPZ- (Figure S2a) and OLA-treated (Figure S2b) groups was discovered for therapeutic, intoxication and fatal-intoxication groups at a low dose (*n* = 8). For each antipsychotic agent, a change in tendency of propionylcarnitine and succinic acid emerged, especially if death occurred (Figure S2).

### 3.7. Sensitivity Evaluation of the Potential-Metabolite Panel in Different Life States at Low Dose

**Figure 5:**
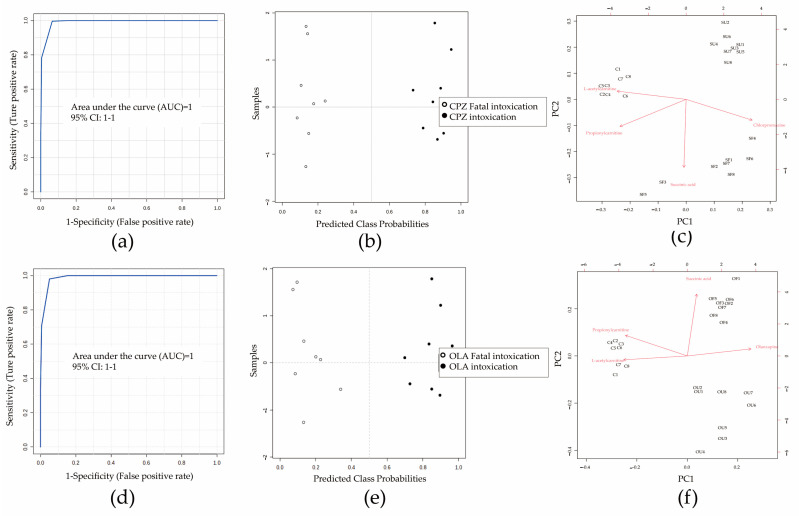
Contributions of three potential metabolites and antipsychotics in different life states at a low dose. ROC curves (**a**,**d**) and predicted class probabilities (**b**,**e**) show the differential performance between fatal intoxications and intoxications caused by CPZ (**a**,**b**; AUC: 1; 95%CI of AUC: 1–1) or OLA (**d**,**e**; AUC: 1; 95%CI of AUC: 1–1). PCA bi-plot showing scatters of samples (black font) from different life states based on three potential metabolites and antipsychotics (red font) profiles. Vector (red straight arrow) projections parallel to between groups show their discriminative power and arrows point in the direction of higher content. Further distinguishing of intoxication with fatal intoxications revealed that CPZ (**c**) or OLA (**f**) (red vectors) contributed less than succinic acid. *n* = 8, respectively. Abbreviations: SU: intoxications caused by CPZ at a low dose; SF: fatal intoxications caused by CPZ at a low dose; OU: intoxications caused by OLA at a low dose; OF: fatal intoxications caused by OLA at a low dose; C: controls.

ROC curves and predicted class probabilities for sensitivity evaluation showed a good classification performance for three potential metabolites between fatal-intoxication and intoxication groups treated by CPZ (AUC: 1; 95%CI of AUC: 1–1; *n* = 8) (Figure 5a,b) or OLA (AUC: 1; 95%CI of AUC: 1–1; *n* = 8) (Figure 5d,e); similar results could be obtained for distinguishing between fatal-intoxication and therapeutic groups (Figure S3). PCA Bi-plots showed a clustering tendency of different life states (normal, intoxication, or fatal intoxication) treated by CPZ (*n* = 8) (Figure 5c) or OLA (*n* = 8) (Figure 5f). Comparison of intoxication groups with fatal-intoxication groups revealed CPZ/OLA to have a weaker distinguishing performance compared with that of succinic acid (Figure 5c,f). A paired sampling method was used between fatal intoxications and intoxications, so metabolite levels were influenced mainly by the state of survival.

### 3.8. Quantitative Analysis of Three Potential Metabolites in Different Life States and at Different Doses

**Figure 6:**
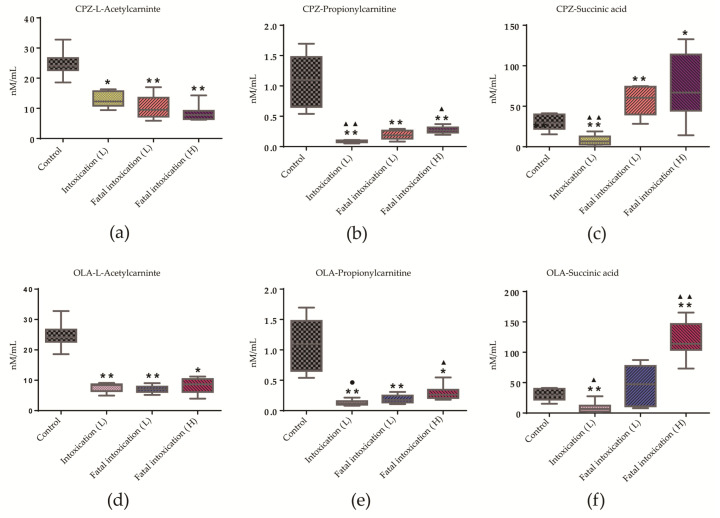
Trends in levels of L-acetylcarnitine, succinic acid, and propionylcarnitine in intoxication and fatal-intoxication groups. (**a**–**c**) CPZ administration. (**d**–**f**) OLA administration. * *p* < 0.05, ** *p* < 0.01 compared with controls. ▲ *p* < 0.05, ▲▲ *p* < 0.01 compared with the fatal-intoxication (low dose) group; ● *p* = 0.055. Significance was determined by Student’s *t*-test or Mann–Whitney U-test. Box plots are expressed as means (horizontal lines), 10 to 90 percentile (boxes), and extent of data (whiskers). *n* = 8. Abbreviations in brackets: L: low dose; H: high dose.

As common differential metabolites, L-acetylcarnitine, succinic acid, and propionylcarnitine showed a similar distribution pattern in intoxication (*n* = 8) and fatal-intoxication (low and high dose, *n* = 8) groups (Figure 6) compared with controls (*n* = 8). L-acetylcarnitine levels were below normal and then unchanged in intoxication and fatal-intoxication groups (Figure 6a,d). Conversely, levels of succinic acid and propionylcarnitine showed significantly differential distributions for fatal-intoxication (low dose) vs. intoxication, or fatal-intoxication (low dose) vs. fatal intoxication (high dose) groups (Figure 6b,c,e,f). Data are summarized as mean ± standard deviation in Table S3.

### 3.9. Stability Evaluation of Three Potential Metabolites in Fresh Plasma

**Figure 7:**
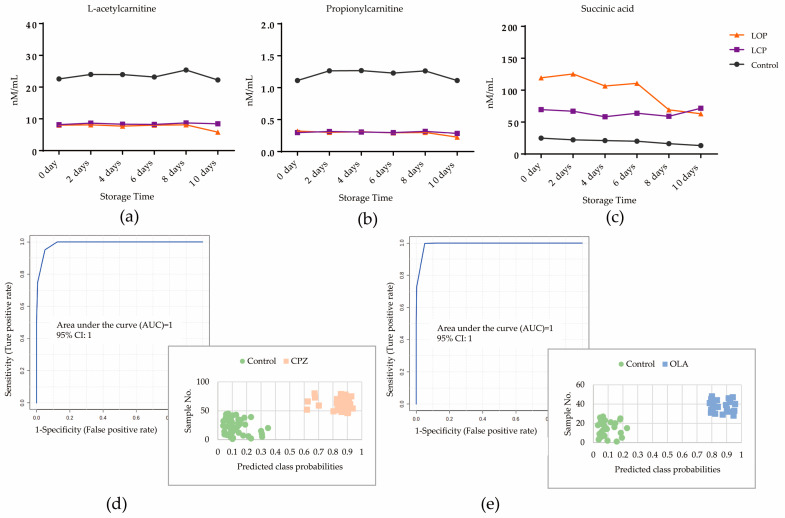
Time-dependent changes in levels of L-acetylcarnitine (**a**), propionylcarnitine (**b**), and succinic acid (**c**) at 4 °C for ≤10 days treated by OLA or CPZ (n_animal_ = 7) at a high dose compared with controls (n_animal_ = 9). Values are expressed as the mean. (**d**) The AUC for prediction performance of fatal intoxication due to CPZ was 1 (95%CI of AUC: 1–1) on day 0. Predicted class probabilities of stored samples within 10 days (bottom right) showed zero misclassifications based on the DCM (Nsample = 7 + 9 on days 2, 4, 6, 8, and 10, respectively). (**e**) The AUC for prediction performance of fatal intoxication due to OLA was 1 (95%CI of AUC: 1–1) on day 0. Predicted class probabilities of stored samples within 6 days (bottom right) showed zero misclassifications based on the DCM (Nsample = 7 + 9 on days 2, 4, and 6). Abbreviations: LCP: lethal poisoning by CPZ at a high dose; LOP: lethal poisoning by CPZ at a high dose.

Animal models comprising fatal intoxication by CPZ (high dose, *n* = 7), fatal intoxication by OLA (high dose, *n* = 7), and controls (*n* = 9, one was excluded as an outlier by PCA) were analyzed in parallel to study the effect of time-dependent changes in three potential metabolites in samples stored at 4 °C for ≤10 days. Trends and deviations in levels of three potential metabolites on days 2, 4, 6, 8, and 10 compared with those on day 0 are shown in Figure 7 and Table S4. In the control group, levels of L-acetylcarnitine and propionylcarnitine were stable within 10 days, whereas the level of succinic acid was reduced and deviated on day 2 compared with day 0. However, three potential metabolites were mostly stable within 10 days in the CPZ group. For the OLA group, levels of L-acetylcarnitine and propionylcarnitine were reduced and deviated on day 10, and the level of succinic acid was reduced and deviated after day 8. The prediction performance of the DCM constructed on three potential metabolites for fatal intoxication by CPZ or OLA on day 0 was 1 (95%CI of AUC: 1–1) (Figure 7d,e) for both. With respect to metabolite decomposition (especially succinic acid), 8/14 cases in the OLA group were misclassified compared with 0/18 in the control group on days 8 and 10, whereas 0 misclassifications were noted in the OLA group on days 2–6 (Figure 7d) and CPZ group on days 2–10 (Figure 7e).

## 4. Discussion

We used MA to: (i) explore changes in metabolite profiles; (ii) screen potential metabolites associated with fatal intoxication by CPZ or OLA. The relative specificity and sensitivity of a classification model comprising L-acetylcarnitine, succinic acid, and propionylcarnitine were verified by comparison with other fatal drug intoxications or CPZ/OLA in different life states at an identical dose. The dynamic trends of three potential metabolites revealed procedural perturbations in metabolic pathways related to fatal intoxication by CPZ or OLA. Importantly, the trend in change for each metabolite may be not unique to fatal intoxication by CPZ or OLA; however, their united and dynamic features could be helpful if combined with drug information for interpretation and classification of fatal intoxication by CPZ or OLA.

### 4.1. Availability of Potential Metabolites by MA

Methods of data normalization and transformation can have a substantial impact on results. Metaboanalyst software introduces various methods to achieve data normalization. Autoscaling and logarithmic transformation are the standardization methods recommended by application trainers when conducting analysis of metabolomic data from blood. Compared with other data-scaling methods used commonly (e.g., Parto scaling), autoscaling can reduce within-group bias and achieve a greater degree of normal distribution of data, which is beneficial for subsequent PCA and OPLS-DA.

Untargeted MA showed a similar metabolite profile in animals treated with typical and atypical antipsychotics (Figure 2) at a high dose. L-acetylcarnitine, succinic acid, and propionylcarnitine were primary endogenous differential metabolites and related mechanistically to fatal intoxication. Their contents were relatively sensitive to some cases of antipsychotic-associated fatal intoxication and could be distinguished further from intoxication (Figure 5). Hence, they could be used as additional evidence for ensuring accurate judgement of fatal intoxication. L-carnitine is a differential metabolite that participates mainly in oxidation of fatty acids. However, simple metabolite models are more robust and cost-effective and less prone to over-fitting, so L-carnitine (coupled with L-acetylcarnitine) was discarded as redundant according to K-means clustering [22].

The relative specificity of the DCM (Figure 4) may have been influenced by pharmacodynamic properties. Except for therapeutic drugs involved in our study, Ning et al. reported that abnormal metabolic pathways for the oxidation of fatty acids and TCA cycle were involved in the process of heroin abuse [37], indicating that its metabolic profile is similar with that of antipsychotic intoxication to some extent. In the present study, L-acetylcarnitine, propionylcarnitine, and succinic acid were screened as the most differential metabolites involved in the metabolism of fatty acids and TCA cycle in the process of fatal intoxication by antipsychotics. As part of follow-up research, we will also screen for differential metabolites with high explanatory and discriminating powers for exclusion by poisoning by other drug types (e.g., sedative-hypnotic, alcohol, illegal drugs). Screening of the potential metabolites for lethal poisoning by different drug types can provide deeper understanding of their different effects at the metabolome level.

### 4.2. Interpretation of L-Acetylcarnitine, Propionylcarnitine, and Succinic Acid for Fatal Intoxication

As evidence of fatal intoxication, a profile of an unchanged level of L-acetylcarnitine combined with increasing levels of propionylcarnitine and succinic acid different from the normal state may be helpful (Figure 6). Pre mortem levels of these three potential metabolites were important references for better interpretation of intoxication and the cause of death. Notably, if levels of L-acetylcarnitine and propionylcarnitine were below that of the control, a rebound of succinic-acid level could be a sign of fatal intoxication.

L-acetylcarnitine and propionylcarnitine are short-chain acylcarnitines and primarily potentiate the TCA cycle by generating acetyl-coenzyme A (CoA) and propionyl-CoA through the oxidation of fatty acids [38]. Propionyl-CoA also serves as an anaplerotic in the TCA cycle if its carboxylation to methyl malonyl-CoA is converted to succinyl-CoA [39], which is a source of succinic acid. Combined with analysis of metabolite pathways, fatal intoxication by antipsychotic agents is caused first by perturbations in the β-oxidation of fatty acids (as marked by L-acetylcarnitine, propionylcarnitine, and L-carnitine). Then, decompensation of the TCA cycle leads to exacerbation (as marked by propionylcarnitine and succinic acid), which results in fatal dysregulation of the metabolism of arginine and proline (as marked by succinic acid). With the development of “multi-omics”, combination of data on the metabolome and proteome may aid further correlation of these perturbed metabolic pathways for fatal intoxication using antipsychotics with specific toxicity symptoms before death.

### 4.3. Impacts of Decomposition of Potential Metabolites on Prediction of the DCM

As demonstrated by stability studies of three metabolites, decomposition of components weakened the prediction performance of the DCM, especially for succinic acid. For the binary classification model, metabolites with trends close to the cutoff level were misclassified primarily (as demonstrated in the OLA group). In view of these influences, in cases where the deviations in concentrations of succinic acid in the fatal-intoxication group were within 15%, the prediction performance of classification was similar to that of a fresh sample. With regard to the preservation time, temperature was an important factor.

### 4.4. Limitations

Our study had three main limitations. First, due to the capabilities of LC-MS detectors and records of databases, the metabolites we focused upon and could identify were very limited. The second limitation was replacing post mortem samples with preserved samples for stability analysis. The small animals (35–40 g) used in our study could suffer obvious coagulation in a short time after death (5–10 min), and the content of propionylcarnitine was much lower in intoxication groups; therefore, using a small sample volume was difficult to achieve. The third limitation was application transition based on animal studies. Ethnic differences may cause some metabolic pathways in the plasma of CD1 mice to be different from that of the human population. The metabolite sets recorded in SMPD and KEGG databases were based on human metabolic pathways. Several population-based studies have demonstrated abnormalities in the oxidation of fatty acid pathway and dysregulation of L-acetylcarnitine [18,40], which was verified in our study. Our previous results from preliminary experiments also showed that, compared with the pathway for the oxidation of fatty acids, the deviation in the TCA metabolic pathway in blood between CD1 experimental animals and a local healthy population was smaller. This finding provides a basis for applied translational research.

## 5. Conclusions

A metabolite panel comprising L-acetylcarnitine, propionylcarnitine, and succinic acid was constructed by novel use of MS/MS full-scan and MRM detection coupled with chemometric models. The relative specificity and sensitivity of the classification model was verified using other drugs with different pharmacodynamic properties or in different life states at an identical drug dose. A profile of an unchanged level of L-acetylcarnitine combined with increasing levels of propionylcarnitine and succinic acid between fatal-intoxication and intoxication groups revealed procedural perturbations in metabolic pathways related to fatal intoxication by CPZ/OLA. Comprehensive and dynamic changes in these three potential metabolites during survival and after death may provide valuable references for interpreting the cause of fatal intoxication by antipsychotic agents.

## Figures and Tables

**Table 1:** Information of study groups.

Group	Drug (Dose)	Aim
Fatal intoxication and Control	CPZ (H ^a^), OLA (H)	Overall metabolomics analysis; analysis of perturbed pathways; construction of a DCM.
Fatal intoxication and Fatal intoxication	CPZ (H), OLA (H), clozapine (H), perphenazine (H), promethazine (H), estazolam (H)	Specificity evaluation of the panel of potential metabolites.
Fatal intoxication, Intoxication, Therapy, and Control	CPZ (L ^b^ and T ^c^), OLA (L and T)	Dynamic changes of three potential metabolites at different drug levels.
Fatal intoxication, Intoxication, and Control	CPZ (L), OLA (L)	Sensitivity evaluation of the panel of potential metabolites.
Fatal intoxication, Intoxication, and Control	CPZ (H and L), OLA (H and L)	Quantitative analysis of three potential metabolites in different life states and at different doses.
Fatal intoxication and Control	CPZ (H), OLA (H)	Stability analysis of three potential metabolites within 10 days.

Note: ^a^: High dose. Only fatal intoxications were calculated. We chose several folds of LD_50_ for a high dose (which was the lowest lethal drug dose) to use fewer animals based on pre-experimental results. ^b^: Low dose. Fatal intoxications and intoxications were included. We chose 2-fold LD_50_ for CPZ based on pre-experimental results and because the bioavailability of CPZ varies due to first-pass metabolism by the liver. ^c^: Therapeutic dose. The treatment for this animal model was CPZ (twice a day), OLA (once a day) for 2 weeks.

**Table 2:** Differential metabolites after fatal intoxication by CPZ.

Compound Name ^a^	Metabolite Identification			*P* ^d^	Trend ^e^	VIP ^f^
	Identified Levels ^b^	Accurate Mass ^c^	RT ^c^			
Chlorpromazine	1	318.09555	10.888	2.82 × 10^−8^	up	1.783209
L-Acetylcarnitine	1	203.11574	9.1	5.77 × 10^−7^	down	1.687712
Propionylcarnitine	1	217.13124	9.878	5.77 × 10^−7^	down	1.45201
Allantoin	2	158.04388	0.921	7.66 × 10^−7^	up	1.703279
Citric acid	2	192.02698	1.526	0.000002	up	1.69585
L-Carnitine	2	161.10506	0.886	3.2 × 10^−6^	down	1.70532
Docosahexaenoic acid	2	328.24034	14.245	2.46 × 10^−5^	up	1.573368
3-Hydroxymethylglutaric acid	2	162.05278	2.226	2.75 × 10^−5^	up	1.574867
Oxoglutaric acid	2	146.02148	1.526	4.38 × 10^−5^	up	1.561638
Succinic acid	1	118.02652	0.902	8.79 × 10^−5^	up	1.497697
Gluconic acid	2	196.05826	0.888	9.51 × 10^−5^	up	1.462974
Kyotorphin	2	337.1733	8.263	9.51 × 10^−5^	down	1.42069
Threonic acid	2	136.03716	0.911	9.51 × 10^−5^	up	1.473649
Hexadecanedioic acid	2	286.21451	12.576	0.000101	up	1.522403
R-3-Hydroxydecanoic acid	2	188.14122	11.839	0.000115	down	1.388931
Dodecanedioic acid	2	230.15171	10.764	0.000165	down	1.430021
3-Oxotetradecanoic acid	2	242.18822	12.827	0.000329	up	1.445228
Choline	2	103.09959	0.868	0.000348	up	1.382057
N-Acetyl-L-alanine	2	131.05818	2.068	0.000617	up	1.339138
Indolelactic acid	2	205.07378	9.693	0.000747	up	1.352877
L-Lysine	2	146.10545	1.053	0.000747	up	1.399428
2-Octanamidoacetic acid	2	201.13647	10.953	0.001291	up	1.296582
Pipazethate	2	399.1614	9.616	0.001915	up	1.217837
Prostaglandin G2 2-glyceryl Ester	2	442.25679	12.041	0.002218	up	1.263507
Galactitol	2	182.07882	0.89	0.004836	up	1.185343
Indoxyl sulfate	2	213.00958	6.979	0.005944	up	1.133043
Hexanoylglycine	2	173.10519	9.126	0.009866	up	1.086799
L-Valine	2	117.07885	0.926	0.023731	down	1.028871

^a^: The annotation for each compound is based on the HMDB database. ^b^: The identification level of metabolites refers to the Metabolomics Standards Initiative reported by Sumner and coworkers. ^c^: Accurate mass and RT were obtained from the mass spectrometer (Q Exactive) on full-scan mode. ^d^: Calculated by Student’s *t*-test. Test level a = 0.05 and *p* < 0.05 means that the distribution of this metabolite between the fatal-intoxication group (at a high dose) and controls was significant. ^e^: Comparison with controls. ^f^: The VIP value is used to measure the ability of each metabolite to explain fatal intoxication, thereby assisting in the screening of marker metabolites (usually with VIP > 1.0 as the screening criterion).

**Table 3:** Differential metabolites after fatal intoxication by OLA.

Compound Name	Metabolite Identification			*p*	Trend	VIP
	Identified Levels	Accurate Mass	RT			
Olanzapine	1	156.07026	6.742	1.38 × 10^−9^	up	1.874806
L-Acetylcarnitine	1	203.11574	9.1	9.85 × 10^−8^	down	1.763666
Propionylcarnitine	1	217.13124	9.878	3.44 × 10^−7^	down	1.717037
Creatine	2	131.06938	0.918	3.86 × 10^−6^	down	1.726218
L-Carnitine	2	161.10506	0.886	3.41 × 10^−5^	down	1.665984
Hexadecanedioic acid	2	286.21451	12.576	3.91 × 10^−5^	up	1.646536
L-Methionine	2	149.05097	0.983	3.91 × 10^−5^	down	1.678973
L-Tyrosine	2	181.07386	0.967	5.53 × 10^−5^	down	1.573716
3-Oxotetradecanoic acid	2	242.18822	12.827	6.44 × 10^−5^	up	1.608964
4-Hydroxycinnamic acid	2	164.0473	0.96	6.44 × 10^−5^	down	1.558905
2-Octanamidoacetic acid	2	201.13647	10.953	0.000151	up	1.54696
12-Hydroxystearic acid	2	300.26648	13.559	0.000372	up	1.455051
Indolelactic acid	2	205.07378	9.693	0.000372	up	1.489531
Taurine	2	125.01455	0.875	0.000501	down	1.508088
Vigabatrin	2	129.07892	0.757	0.001378	down	1.369638
L-Valine	2	117.07885	0.926	0.001598	down	1.440964
Allantoin	2	158.04388	0.921	0.002728	up	1.353355
Galactitol	2	182.07882	0.89	0.003248	up	1.230641
Gluconic acid	2	196.05826	0.888	0.006513	up	1.160182
Succinic acid	1	118.02652	0.902	0.006513	up	1.34286
Leucyl-Glutamate	2	260.13706	5.911	0.007345	down	1.281477
Threonic acid	2	136.03716	0.911	0.013767	up	1.080708
R-3-Hydroxydecanoic acid	2	188.14122	11.839	0.015148	down	1.103047
LysoPC2055Z,8Z,11Z,14Z,17Z/00	2	541.31669	12.317	0.016543	up	1.095663
Cholic acid	2	408.28784	11.728	0.017701	down	1.137465
Citric acid	2	192.02698	1.526	0.018878	up	1.072548
L-Lysine	2	146.10545	1.053	0.021913	down	1.01019
Hexanoylglycine	2	173.10519	9.126	0.026868	up	1.004025
Dodecanedioic acid	2	230.15171	10.764	0.038024	down	1.02619

## Data Availability

The data presented in this study are available in supplementary material.

## Supplementary Materials

The following supporting information can be downloaded at: https://www.mdpi.com/article/10.3390/metabo12121184/s1, Figure S1: MRM chromatograms of extracts of plasma samples; Figure S2: Trends of three potential metabolites as drug levels increased; Figure S3: Differential performance of a panel of three potential metabolites between fatal-intoxication (low dose) and therapeutic groups; Table S1: Identification information and prediction performance of each differential metabolite; Table S2: Prediction results of new samples; Table S3: Distribution of potential metabolites in different life states and at different dose; Table S4: Deviation of potential biomarker levels in fresh plasma after 10-day storage at 4 °C.

## References

[1] Gummin D.D., Mowry J.B., Beuhler M.C., Spyker D.A., Bronstein A.C., Rivers L.J., Pham N.P.T., Weber J. (2021). 2020 Annual report of the American Association of Poison Control Centers’ National Poison Data System (NPDS): 38th annual report. Clin. Toxicol..

[2] Toft S., Horwitz H., Dalhoff K.P. (2017). Long-term mortality after poisoning with antipsychotics. Clin. Toxicol..

[3] Schreinzer D., Frey R., Stimpfl T., Vycudilik W., Berzlanovich A., Kasper S. (2001). Different fatal toxicity of neuroleptics identified by autopsy. Eur. Neuropsychopharmacol. J. Eur. Coll. Neuropsychopharmacol..

[4] Okumura Y., Sakata N., Takahashi K., Nishi D., Tachimori H. (2017). Epidemiology of overdose episodes from the period prior to hospitalization for drug poisoning until discharge in Japan: An exploratory descriptive study using a nationwide claims database. J. Epidemiol..

[5] Pan M., Wang X., Zhao Y., Liu W., Xiang P. (2019). A retrospective analysis of data from forensic toxicology at the Academy of Forensic Science in 2017. Forensic Sci. Int..

[6] Mowry J.B., Spyker D.A., Cantilena L.R., McMillan N., Ford M. (2014). 2013 annual report of the American Association of Poison Control Centers’ National Poison Data System (NPDS): 31st annual report. Clin. Toxicol..

[7] Solmi M., Murru A., Pacchiarotti I., Undurraga J., Veronese N., Fornaro M., Stubbs B., Monaco F., Vieta E., Seeman M.V. (2017). Safety, tolerability, and risks associated with first- and second-generation antipsychotics: A state-of-the-art clinical review. Ther. Clin. Risk Manag..

[8] Saar E., Gerostamoulos D., Drummer O.H., Beyer J. (2012). Assessment of the stability of 30 antipsychotic drugs in stored blood specimens. Forensic Sci. Int..

[9] DRUGBANK online. https://go.drugbank.com/drugs/DB00477.

[10] Schulz M., Schmoldt A., Andresen-Streichert H., Iwersen-Bergmann S. (2020). Revisited: Therapeutic and toxic blood concentrations of more than 1100 drugs and other xenobiotics. Crit. Care.

[11] Nedahl M., Johansen S., Linnet K. (2018). Reference brain and blood concentrations of olanzapine in postmortem cases. J. Anal. Toxicol..

[12] Smith M.P., Bluth M.H. (2016). Forensic toxicology: An introduction. Clin. Lab. Med..

[13] Beck S.C., Wilding T., Buka R.J., Baretto R.L., Huissoon A.P., Krishna M.T. (2019). Biomarkers in human anaphylaxis: A critical appraisal of current evidence and perspectives. Front. Immunol..

[14] Wishart D.S. (2019). Metabolomics for investigating physiological and pathophysiological processes. Physiol. Rev..

[15] Araújo A.M., Carvalho F., Guedes de Pinho P., Carvalho M. (2021). Toxicometabolomics: Small molecules to answer big toxicological questions. Metabolites.

[16] Steuer A.E., Brockbals L., Kraemer T. (2019). Metabolomic strategies in biomarker research—New approach for indirect identification of drug consumption and sample manipulation in clinical and forensic toxicology?. Front. Chem..

[17] Klingerman C.M., Stipanovic M.E., Bader M., Lynch C.J. (2014). Second-generation antipsychotics cause a rapid switch to fat oxidation that is required for survival in C57BL/6J mice. Schizophr. Bull..

[18] Burghardt K.J., Evans S.J., Wiese K.M., Ellingrod V.L. (2015). An untargeted metabolomics analysis of antipsychotic use in bipolar disorder. Clin. Transl. Sci..

[19] Paredes R.M., Quinones M., Marballi K., Gao X., Valdez C., Ahuja S.S., Velligan D., Walss-Bass C. (2014). Metabolomic profiling of schizophrenia patients at risk for metabolic syndrome. Int. J. Neuropsychopharmacol..

[20] Meister R., von Wolff A., Mohr H., Härter M., Nestoriuc Y., Hölzel L., Kriston L. (2016). Comparative safety of pharmacologic treatments for persistent depressive disorder: A systematic review and network meta-analysis. PLoS ONE.

[21] Jordan K., Wild T.S.N., Fromberger P., Müller I., Müller J.L. (2019). Are there any biomarkers for pedophilia and sexual child abuse? A review. Front. Psychiatry.

[22] Chong J., Wishart D.S., Xia J. (2019). Using MetaboAnalyst 4.0 for comprehensive and integrative metabolomics data analysis. Curr. Protoc. Bioinform..

[23] Wlodarczyk B.J., Ogle K., Lin L.Y., Bialer M., Finnell R.H. (2015). Comparative teratogenicity analysis of valnoctamide, risperidone, and olanzapine in mice. Bipolar Disord..

[24] Yen-Koo H.C., Davis D.A., Balazs T. (1985). Inhibition of dopaminergic agonist-induced gnawing behavior by neuroleptic drugs in mice. Drug Chem. Toxicol..

[25] Yen-Koo H.C., Balazs T. (1980). Detection of dopaminergic supersensitivity induced by neuroleptic drugs in mice. Drug Chem. Toxicol..

[26] DRUGBANK Online. https://go.drugbank.com/drugs/DB01069.

[27] Coccurello R., Caprioli A., Ghirardi O., Conti R., Ciani B., Daniele S., Bartolomucci A., Moles A. (2006). Chronic administration of olanzapine induces metabolic and food intake alterations: A mouse model of the atypical antipsychotic-associated adverse effects. Psychopharmacology.

[28] Krsiak M. (1979). Effects of drugs on behaviour of aggressive mice. Br. J. Pharmacol..

[29] Andreev-Andrievskiy A., Popova A., Lagereva E., Osipov D., Berkut A., Grishin E., Vassilevski A. (2017). Pharmacological analysis of Poecilotheria spider venoms in mice provides clues for human treatment. Toxicon Off. J. Int. Soc. Toxinol..

[30] Xia G. (2019). Dietary methionine influences therapy in mouse cancer models and alters human metabolism. Nature.

[31] Roy C., Tremblay P.Y., Bienvenu J.F., Ayotte P. (2016). Quantitative analysis of amino acids and acylcarnitines combined with untargeted metabolomics using ultra-high performance liquid chromatography and quadrupole time-of-flight mass spectrometry. J. Chromatogr. B Analyt. Technol. Biomed. Life Sci..

[32] Chen Y., Zhou Z., Yang W., Bi N., Xu J., He J., Zhang R., Wang L., Abliz Z. (2017). Development of a data-independent targeted metabolomics method for relative quantification using liquid chromatography coupled with tandem mass spectrometry. Anal. Chem..

[33] Yin P., Xu G. (2014). Current state-of-the-art of nontargeted metabolomics based on liquid chromatography-mass spectrometry with special emphasis in clinical applications. J. Chromatogr. A.

[34] Zhang K., Zhang A., Liu R., Zhang H., Lin H., Zhang P., Huang P., Wang Z. (2020). Identifying muscle hemorrhage in rat cadavers with advanced decomposition by FT-IR microspectroscopy combined with chemometrics. Leg. Med..

[35] Sun Y.C., Han S.C., Yao M.Z., Liu H.B., Wang Y.M. (2020). Exploring the metabolic biomarkers and pathway changes in crucian under carbonate alkalinity exposure using high-throughput metabolomics analysis based on UPLC-ESI-QTOF-MS. RSC Adv..

[36] Sumner L.W., Amberg A., Barrett D., Beale M.H., Beger R., Daykin C.A., Fan T.W., Fiehn O., Goodacre R., Griffin J.L. (2007). Proposed minimum reporting standards for chemical analysis Chemical Analysis Working Group (CAWG) Metabolomics Standards Initiative (MSI). Metabolomics.

[37] Ning T., Leng C., Chen L., Ma B., Gong X. (2018). Metabolomics analysis of serum in a rat heroin self-administration model undergoing reinforcement based on (1)H-nuclear magnetic resonance spectra. BMC Neurosci..

[38] Norris M.K., Scott A.I., Sullivan S., Chang I.J., Lam C., Sun A., Hahn S., Thies J.M., Gunnarson M., McKean K.N. (2021). Tutorial: Triheptanoin and nutrition management for treatment of long-chain fatty acid oxidation disorders. JPEN J. Parenter. Enter. Nutr..

[39] Di Emidio G., Rea F., Placidi M., Rossi G., Cocciolone D., Virmani A., Macchiarelli G., Palmerini M.G., D’Alessandro A.M., Artini P.G. (2020). Regulatory functions of L-carnitine, acetyl, and propionyl L-carnitine in a PCOS mouse model: Focus on antioxidant/antiglycative molecular pathways in the ovarian microenvironment. Antioxidants.

[40] Cao B., Jin M., Brietzke E., McIntyre R.S., Wang D., Rosenblat J.D., Ragguett R.M., Zhang C., Sun X., Rong C. (2019). Serum metabolic profiling using small molecular water-soluble metabolites in individuals with schizophrenia: A longitudinal study using a pre-post-treatment design. Psychiatry Clin. Neurosci..

