# Supplementary material for: Dynamic Changes in Plasma Metabolic Profiles Reveal a Potential Metabolite Panel for Interpretation of Fatal Intoxication by Chlorpromazine or Olanzapine in Mice
Source: Metabolites. 2022 Nov 27;12(12):1184. doi: 10.3390/metabo12121184 (PMC9782175; doi:10.3390/metabo12121184)
Supplement: Supplementary file 1 [file metabolites-12-01184-s001.zip › metabolites-1999827-supplementary.pdf]

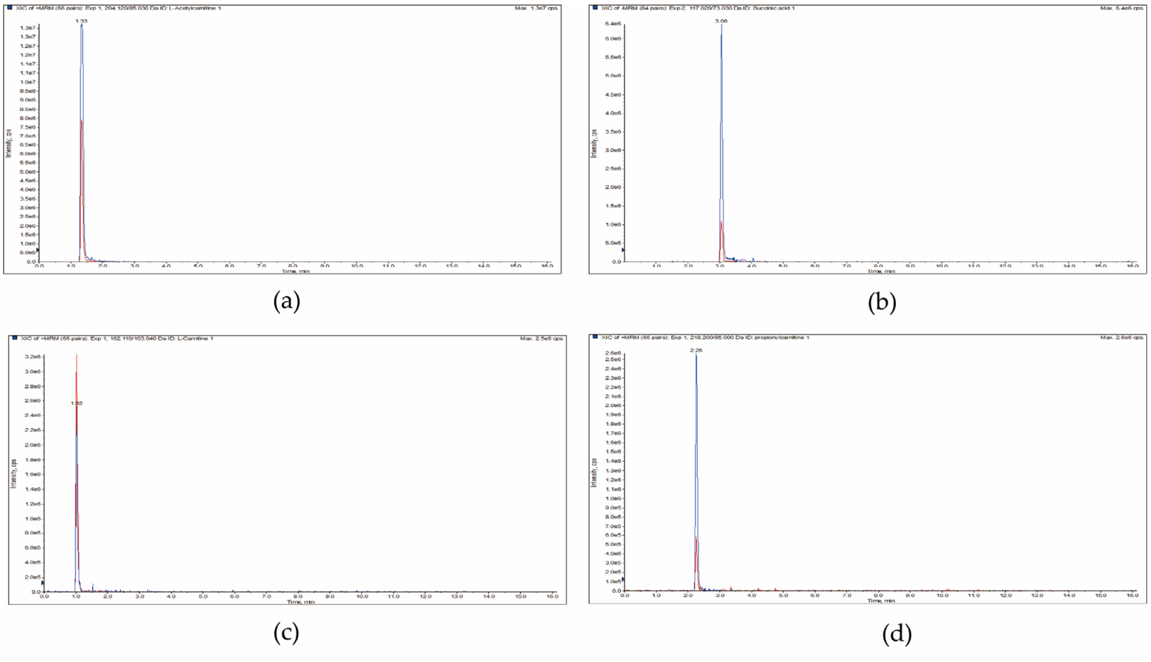

Figure S1. MRM chromatograms of extracts of plasma samples. (a) L-acetylcarnitine, (b) succinic acid, (c) L-carnitine, and (d) propionylcarnitine.

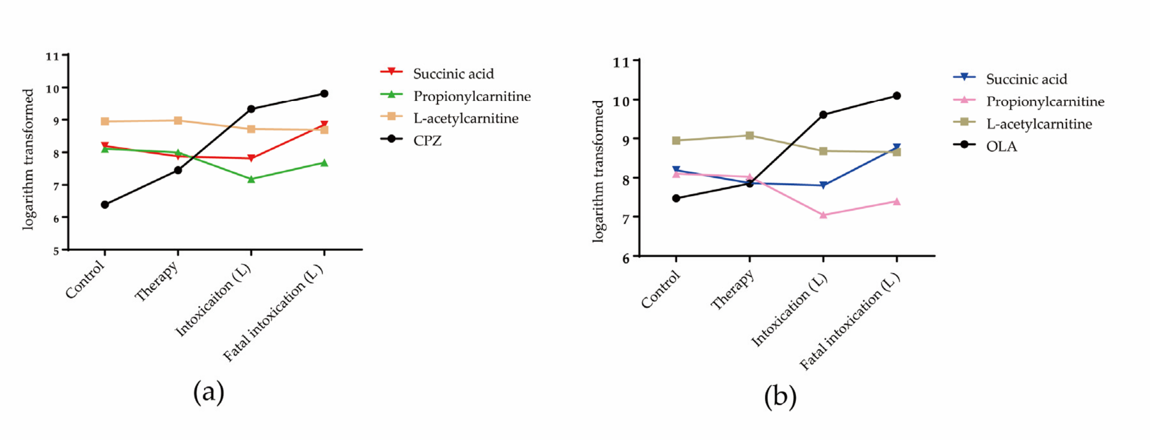

Figure S2. Trends of three potential metabolites as drug levels increased. As the drug level increased, the trend pattern of levels of three potential metabolites between CPZ (a) and OLA (b) groups were similar; a change in tendency of propionylcarnitine and succinic acid emerged, especially if death occurred. Data are mean values. n = 8, respectively.

Note: For relative quantitative analysis, the abundance of CPZ/OLA in controls was not zero because of processing of missing values by Compound Discoverer.

Abbreviations in brackets: L: low dose.

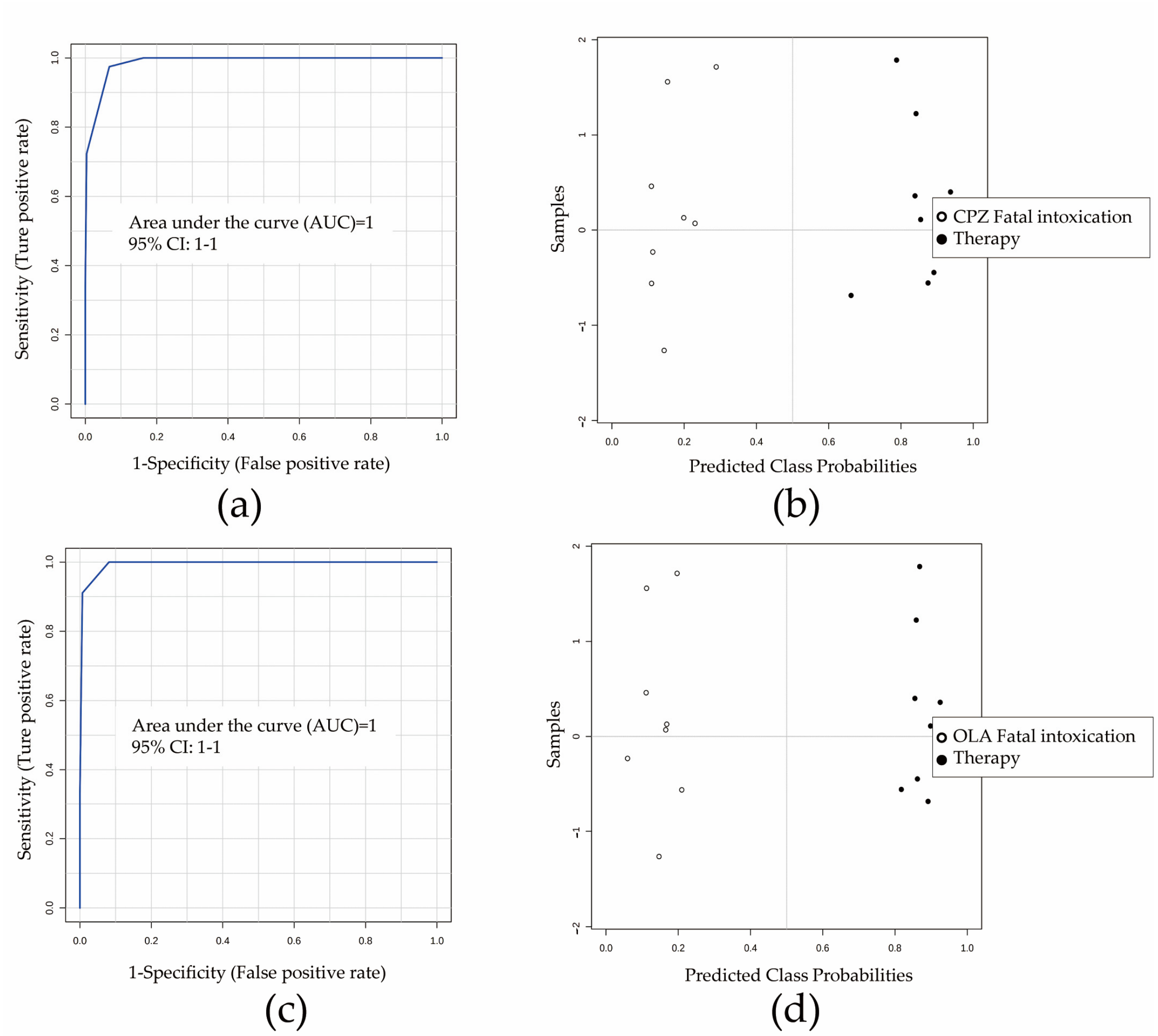

Figure S3. Differential performance of the panel of three potential metabolites between fatal-intoxication (low dose) and therapeutic groups. ROC curves (a, c) and predicted class probabilities (b, d) show the differential performance between fatal-intoxication groups and therapeutic groups treated by CPZ (a, b; AUC: 1; 95%CI of AUC: 1–1) or OLA (c, d; AUC: 1; 95%CI of AUC: 1–1). n = 8, respectively.

Table S1. Identification information and prediction performance of each differential metabolite.

Identified compound	RT (min)	Parent type	Q1 (amu)	Q3 (amu)	Declustering potential (V)	Collision energy (eV)	AUC
**L-Acetylcarnitine**	1.35	$[M+H]^+$	204.12	145.2	70	16.88	1.0
			204.12	85.1	70	25.67	
**L-Carnitine**	0.98	$[M+H]^+$	162.11	103.04	80	14.59	1.0
			162.11	60.08	80	14.59	
**Propionylcarnitine**	2.29	$[M+H]^+$	218.2	85	80	25	0.98
			218.2	159.2	80	18	
**Succinic acid**	3.05	$[M-H]^-$	117.02	73.03	-50	-15	0.95
			117.02	99.01	-70	-15	
**Propionylcarnitine-(N-methyl-d3) (IS)**	2.3	$[M+H]^+$	221.2	85.1	80	25	-
			221.2	159.2	80	18	
**Succinic acid-2,2,3,3-d4 (IS)**	3.02	$[M-H]^-$	121.1	77.1	-60	-15.06	-
			121.1	102.1	-80	-16.2	
**L-Acetylcarnitine-(N-methyl-d3) (IS)**	1.37	$[M+H]^+$	207.2	145.2	110	16.51	-
			207.2	85.2	100	24.59	

Table S2. Prediction results of new samples.

Sample No.	Probability	Class
1	0.98	Antipsychotics intoxication
2	0.94	Antipsychotics intoxication
3	0.93	Antipsychotics intoxication
4	0.96	Antipsychotics intoxication
5	0.94	Control
6	0.88	Control

Table S3. Distribution of potential metabolites in different life states and at different dose.

Drug-compounds (nM/mL)	Intoxication (low dose, n=8)	Fatal intoxication (low dose, n=8)	Fatal intoxication (high dose, n=8)	Control (n=8)
**CPZ-L-Acetylcarnitine**	12.91±2.646	10.51±3.831	7.805 (6.423-9.173)	24.77±4.128
**OLA-L-Acetylcarnitine**	7.755±1.493	7.04±1.269	8.289±2.521	
**CPZ-Propionylcarnitine**	0.0857±0.019	0.190±0.0738	0.274±0.058	1.089±0.421
**OLA-Propionylcarnitine**	0.1300±0.0425	0.189±0.067	0.262 (0.208-0.344)	
**CPZ-Succinic acid**	7.864±6.185	56.99±17.88	74.69±41.58	29.6±9.595
**OLA-Succinic acid**	6.674 (2.073-12.29)	45.47±33.95	119.2±29.35	

Table S4. Deviation of potential biomarker levels in fresh plasma after 10-day storage at 4°C.

Time Group	L-acetylcarnitine (nM/mL)			Propionylcarnitine (nM/mL)			Succinic acid (nM/mL)		
	Control	CPZ	OLA	Control	CPZ	OLA	Control	CPZ	OLA
0 day	24.8	8.181	8.025	1.139	0.278	0.2994	27.67	69.64	119.3
2 days	24.67 (0.5%)	8.656 (5.8%)	8.123 (1.2%)	1.152 (1.1%)	0.2942 (5.8%)	0.2807 (6.2%)	23.01 (16.8%) *	67.15 (3.6%)	125.4 (5.1%)
4 days	24.42 (1.5%)	8.325 (1.8%)	7.707 (4.0%)	1.148 (0.8%)	0.2853 (2.6%)	0.2905 (2.9%)	22.3 (19.4%) *	58.4 (16.1%) *	106.5 (10.7%)
6 days	23.42 (5.5%)	8.254 (0.9%)	8.012 (0.1%)	1.123 (1.4%)	0.2777 (0.1%)	0.2732 (8.7%)	21.23 (23.2%) *	63.89 (8.2%)	110.7 (7.2%)
8 days	25.9 (4.4%)	8.728 (6.7%)	8.124 (1.2%)	1.147 (0.7%)	0.2956 (6.3%)	0.2802 (6.4%)	16.45 (40.5%) *	59.07 (15.2%) *	69.27 (41.9%) *
10 days	22.71 (8.4%)	8.438 (3.1%)	5.817 (27.5%) *	1.037 (9.0%)	0.2658 (4.3%)	0.2106 (29.7%) *	13.52 (51.1%) *	71.7 (3.0%)	63.06 (47.1%) *

Note: Values in brackets represents deviation (absolute value) calculated as: (levels of day (2-10) – levels of day0) divided by levels of day 0. Where component levels were showed as mean value.
*, With deviations >15%.
